# Characterization of *HMGA2* variants expands the spectrum of Silver-Russell syndrome

**DOI:** 10.1172/jci.insight.169425

**Published:** 2024-03-22

**Authors:** Avinaash V. Maharaj, Emily Cottrell, Thatchawan Thanasupawat, Sjoerd D. Joustra, Barbara Triggs-Raine, Masanobu Fujimoto, Sarina G. Kant, Danielle van der Kaay, Agnes Clement-de Boers, Alice S. Brooks, Gabriel Amador Aguirre, Irene Martín del Estal, María Inmaculada Castilla de Cortázar Larrea, Ahmed Massoud, Hermine A. van Duyvenvoorde, Christiaan De Bruin, Vivian Hwa, Thomas Klonisch, Sabine Hombach-Klonisch, Helen L. Storr

**Affiliations:** 1 Centre for Endocrinology, William Harvey Research Institute, QMUL, London, United Kingdom.; 2 Department of Human Anatomy and Cell Science, University of Manitoba, Winnipeg, Manitoba, Canada.; 3 Division of Paediatric Endocrinology, Department of Paediatrics, Willem-Alexander Children’s Hospital, Leiden University Medical Centre, Leiden, Netherlands.; 4 Department of Biochemistry and Medical Genetics, University of Manitoba, Winnipeg, Manitoba, Canada.; 5 Cincinnati Center for Growth Disorders, Division of Endocrinology, Cincinnati Children’s Hospital Medical Center, Department of Pediatrics, University of Cincinnati College of Medicine, Cincinnati, USA.; 6 Division of Paediatric Endocrinology, Department of Paediatrics, Erasmus University Medical Centre, Sophia Children’s Hospital, Rotterdam, Netherlands.; 7 Department of Paediatrics, Juliana Children’s Hospital/Haga Teaching Hospital, The Hague, Netherlands.; 8 Department of Clinical Genetics, Erasmus MC University Medical Center, Rotterdam, Netherlands.; 9 CITES - Escuela Nacional de Medicina, TEC de Monterrey, Monterrey, Nuevo León, Mexico.; 10 Department of Paediatrics and Child Health, HCA Healthcare UK, London, United Kingdom.; 11 Laboratory for Diagnostic Genome analysis (LDGA), Department of Clinical Genetics, Leiden University Medical Centre, Leiden, Netherlands.; 12 Department of Pathology, and; 13 Department of Medical Microbiology and Infectious Diseases, University of Manitoba, Winnipeg, Manitoba, Canada.

**Keywords:** Endocrinology, Genetics, Growth factors, Molecular genetics

## Abstract

Silver-Russell syndrome (SRS) is a heterogeneous disorder characterized by intrauterine and postnatal growth retardation. *HMGA2* variants are a rare cause of SRS and its functional role in human linear growth is unclear. Patients with suspected SRS negative for 11p15LOM/mUPD7 underwent whole-exome and/or targeted-genome sequencing. Mutant HMGA2 protein expression and nuclear localization were assessed. Two *Hmga2*-knockin mouse models were generated. Five clinical SRS patients harbored *HMGA2* variants with differing functional impacts: 2 stop-gain nonsense variants (c.49G>T, c.52C>T), c.166A>G missense variant, and 2 frameshift variants (c.144delC, c.145delA) leading to an identical, extended-length protein. Phenotypic features were highly variable. Nuclear localization was reduced/absent for all variants except c.166A>G. Homozygous knockin mice recapitulating the c.166A>G variant (*Hmga2*^K56E^) exhibited a growth-restricted phenotype. An *Hmga2^Ter76^*-knockin mouse model lacked detectable full-length Hmga2 protein, similarly to patient 3 and 5 variants. These mice were infertile, with a pygmy phenotype. We report a heterogeneous group of individuals with SRS harboring variants in *HMGA2* and describe the first *Hmga2* missense knockin mouse model (*Hmga2*^K56E^) to our knowledge causing a growth-restricted phenotype. In patients with clinical features of SRS but negative genetic screening, *HMGA2* should be included in next-generation sequencing testing approaches.

## Abstract 2

In undiagnosed patients with clinical features of Silver-Russell syndrome, but negative molecular/genetic analysis, HMGA2 testing should be considered, particularly in those presenting with microcephaly.

## Introduction

Silver-Russell syndrome (SRS, OMIM 180860) is a genetically heterogeneous disorder characterized by intrauterine and postnatal growth retardation, relative macrocephaly, protruding forehead, feeding difficulties, and body asymmetry (1). SRS is a clinical diagnosis based on phenotypic criteria. The Netchine-Harbison clinical scoring system (NH-CSS), the only comprehensive screening tool for SRS, mandates the presence of 4 out of 6 classical features, including relative macrocephaly and prominent forehead, which are requisite for establishing a clinical diagnosis (1). Despite low specificity, the NH-CSS has a high negative predictive value for discounting non-SRS small for gestation age (SGA) children, provided strict adherence to diagnostic inclusion criteria. Wide phenotypic variability exists and additional associated SRS features include triangular face, fifth finger clinodactyly, shoulder dimples, micrognathia, low muscle mass, developmental delay, and hypoglycemia (1).

Molecular testing confirms SRS in 60%–70% of patients. Hypomethylation of the imprinted *H19/IGF2* domain of chromosome 11p15 (11p15LOM) and maternal uniparental disomy of chromosome 7 (mUPD7) are identified in 50%–60% and 10% of SRS cases, respectively (2). The genetic etiology remains unknown in approximately 30% of clinical SRS cases (1). Other rarer genetic causes include mUPD20 and monogenic defects in imprinted genes *IGF2*, *CDKN1C*, and *PLAG1* (3). The non-imprinted high-mobility group AT-hook 2 (*HMGA2*) (NCBI gene ID: 8091) gene is associated with human height (4) and defects in *HMGA2*, including microdeletions of chromosome 12q14, were recently identified as rare monogenic causes of SRS (5–10).

HMGA2 belongs to a family of small, high-mobility group chromatin-associated proteins characterized by the presence of 3 AT-hook domains that interact with DNA (11). Upon binding to chromatin, the AT-hook domains modify the chromatin architecture to facilitate binding of transcription factors to DNA, thereby influencing gene transcription (12). HMGA2, an important regulator of cell growth, apoptosis, and cell differentiation, is highly expressed in embryonic tissue but largely undetectable in most adult tissues (13). It is overexpressed in a variety of benign and malignant tumors (14–16) and promotes tumorigenesis via multiple mechanisms. These include increased malignant cell proliferation (17–19), enhanced epithelial-mesenchymal transition (20–23) and tissue invasion (24, 25), maintenance of genomic stability (26–30), attenuation of apoptosis (26–31), and promotion of therapeutic resistance (32–36).

Common *HMGA2* variants adjacent to the 3′UTR region have been strongly associated (*P* < 1 × 10^−10^) with childhood and adult final height in several genome-wide association studies (4, 37–39). Several *HMGA2* polymorphisms have been postulated to contribute to idiopathic short stature (6). To date, 9 pathogenic *HMGA2* variants in 11 patients, including 1 in a sibling pair, have been reported in individuals with short stature and SRS features scoring 4 or 5 out of 6 NH-CSS criteria (5, 9, 10, 40, 41). Interestingly, 3 lacked classic macrocephaly vital for a clinical diagnosis (9), suggesting a phenotypic spectrum that differs from classical SRS. Loss of *Hmga2* in 2 different transgenic mouse models produces growth failure and a pygmy phenotype despite sufficient growth hormone (GH) levels. This suggests that loss of one allele impacts normal growth physiology (42, 43).

Despite the strong evidence for the crucial role of *HMGA2* in growth modulation (43–49), underlying regulatory mechanisms of HMGA2 in human linear growth remain unclear. We aimed to confirm the pathogenicity of 5 rare variants occurring in different critical regions of the *HMGA2* gene. Knockin mice homozygous for *Hmga2*^K56E^ carrying the new missense mutation, c.166A>G (p.Lys56Glu), located in the linker 2 region of *HMGA2* demonstrated that a single amino acid change located outside of AT-hook domains can modulate growth in mice. We also showed that expression of an N-terminal fragment of *Hmga2* devoid of AT-hook 3 and the C-terminus results in a pygmy phenotype characteristic of *Hmga2* gene knockout. Our study expands the clinical spectrum of this human growth disorder and provides insights into the fundamental functional role of *HMGA2* as a gene implicated in growth.

## Results

### Clinical and genetic details of the HMGA2 variant probands.

We identified 5 heterozygous *HMGA*2 variants in 5 probands with pre- and postnatal growth failure (Table 1).

Patient 1, a female of South Asian ethnicity, was born SGA with intrauterine growth restriction noted on prenatal surveillance scans. Postnatally, the patient was growth restricted with failure to thrive, feeding difficulties, and mild developmental delay. At 5.8 years of age, examination revealed a triangular face, high-pitched voice, and high-arched palate. A maternally inherited heterozygous *HMGA2* variant, g.66221835A>G, c.166A>G (p.Lys56Glu), was identified and segregated with maternal height (–3.5 SDS).

Patient 2, a Mexican female, was referred at 6 months of age for postnatal growth failure. In utero growth restriction was noted from 29 weeks of gestation, with growth curves below the third centile. Genetic testing identified a heterozygous *HMGA2* frameshift variant, g.66221814del, c.145delA (p.Arg49Glyfs*117). Both parents were of normal stature (–1.2 SDS and +0.1 SDS) and are awaiting genetic testing.

Patient 3, a Dutch female, presented with a history of intrauterine growth restriction and postnatal growth failure. At 3 years of age, the patient was short with a triangular face and relatively large forehead, although head circumference was –2.0 SDS. Genetic testing identified a heterozygous *HMGA2*-truncating variant, g.66219102C>T, c.52C>T (p.Gln18*). This variant was inherited from the patient’s mother who was short (height –3.7 SDS), with similar facial gestalt.

Patient 4, a Dutch female, was born at term with a birth weight of –1.9 SDS and progressive postnatal growth failure. She presented at the age of 6 months with feeding difficulties (necessitating short-term tube feeding), gastro-oesophageal reflux, and failure to thrive. At age 7.5 years, physical examination revealed frontal bossing, mid-facial hypoplasia, and a high-pitched voice. Genetic testing identified a heterozygous *HMGA2* frameshift variant, g.66221813del, c.144delC (p.Arg49Glyfs*117). Genetic testing of biological parents was not possible, although maternal stature was normal (–1.1 SDS).

Patient 5, a Dutch female, was born at term and SGA. At the age of 3 years, she was growth restricted and underweight, although no feeding difficulties were noted. Genetic testing identified a maternally inherited heterozygous truncating variant, g.66219099G>T, c.49G>T (p.Gly17*), in *HMGA2*. There was maternal short stature (–3.7 SDS) following a history of being SGA (birth weight –3.8 SDS). Paternal stature was normal (–0.4 SDS).

### HMGA2 gene variants identified in probands.

**Figure 1:**
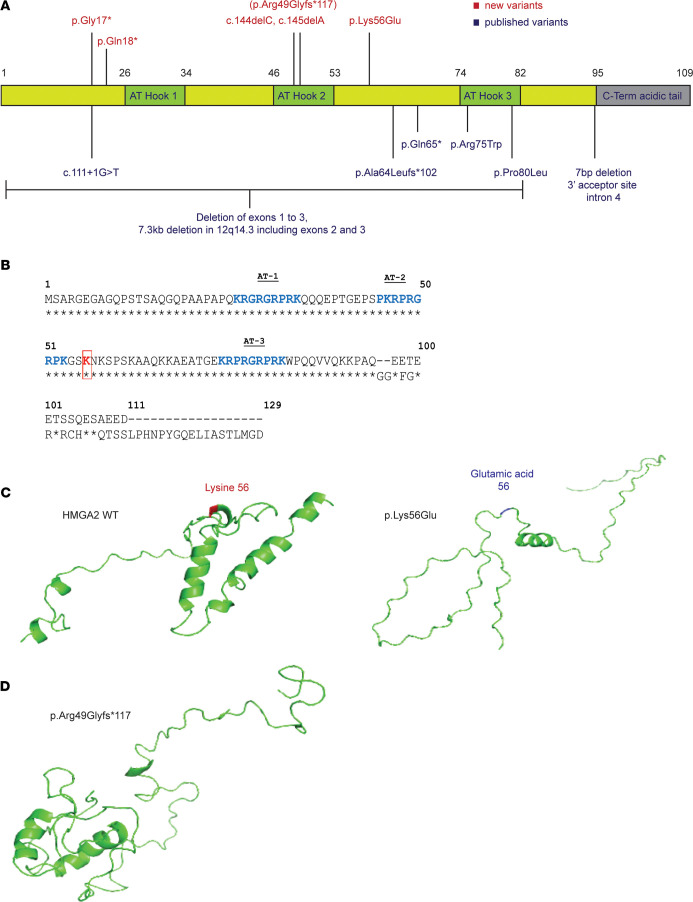
Domain topology and structure of *HMGA2* variants. (**A**) *HMGA2* variants identified in 5 probands are shown in red above the schematic *HMGA2* structure. The functional domains illustrated (AT- hooks 1 to 3) are critical DNA-binding motifs, with variants occurring prior to AT-hook 3 hypothesized to have the most deleterious impact on protein function. The genotype-phenotype correlation is, however, tenuous, with previously reported variants (blue) ranging from single amino acid substitutions to large deletions (9, 10, 40, 41). (**B**) Human HMGA2 and murine Hmga2 protein sequences show a high degree of amino acid identity and conservation of the specific lysine (K) 56 residue (highlighted in red) altered by the p.Lys56Glu variant. (**C**) Replacement of lysine with glutamic acid at amino acid position 56 appears to cause conformational changes to HMGA2, particularly to the C-terminal region. (**D**) Variants c.144delC and c.145delA both give rise to an identical elongated protein p.Arg49Glyfs*117, predicted to result in misfolding of critical DNA-binding domains.

Details of *HMGA2* gene variants are shown in Table 2 and Figure 1A. All 5 variants identified in probands were not previously listed in gnomAD. Of particular interest was the missense heterozygous variant c.166A>G (p.Lys56Glu), harbored in patient 1. Only 2 other missense variants have been reported, both located within the AT-hook 3 domain. The HMGA2 p.Lys56Glu missense variant, assigned a, combined annotation–dependent depletion(CADD) score of 27.2 and predicted “disease-causing” by MutationTaster, was located in a critically important and highly conserved region of *HMGA2* adjacent to the second AT-hook (Figure 1B). Using the IntFOLD computational platform, modeling of the missense variant with a positively charged lysine (K) at position 56 replaced with a negatively charged glutamic acid (E) suggested an overall conformational change to HMGA2 (Figure 1C).

Both of the variants identified in patients 2 and 4 resulted in an identical mutant transcript leading to an extended protein longer than wild-type (WT) HMGA2 that contains an intact AT-hook 1, a severely truncated AT-hook 2, and no AT-hook 3 (Figure 1D). These altered sequences are not predicted to possess full WT function, but may retain some residual activity. The nonsense variants identified in patients 3 (c.52C>T, p.Gln18*) and 5 (c.49G>T, p.Gly17*) are predicted to result in premature stop codons and are likely degraded by nonsense-mediated mRNA decay.

### HMGA2 variant in vitro expression and nuclear localization.

**Figure 2:**
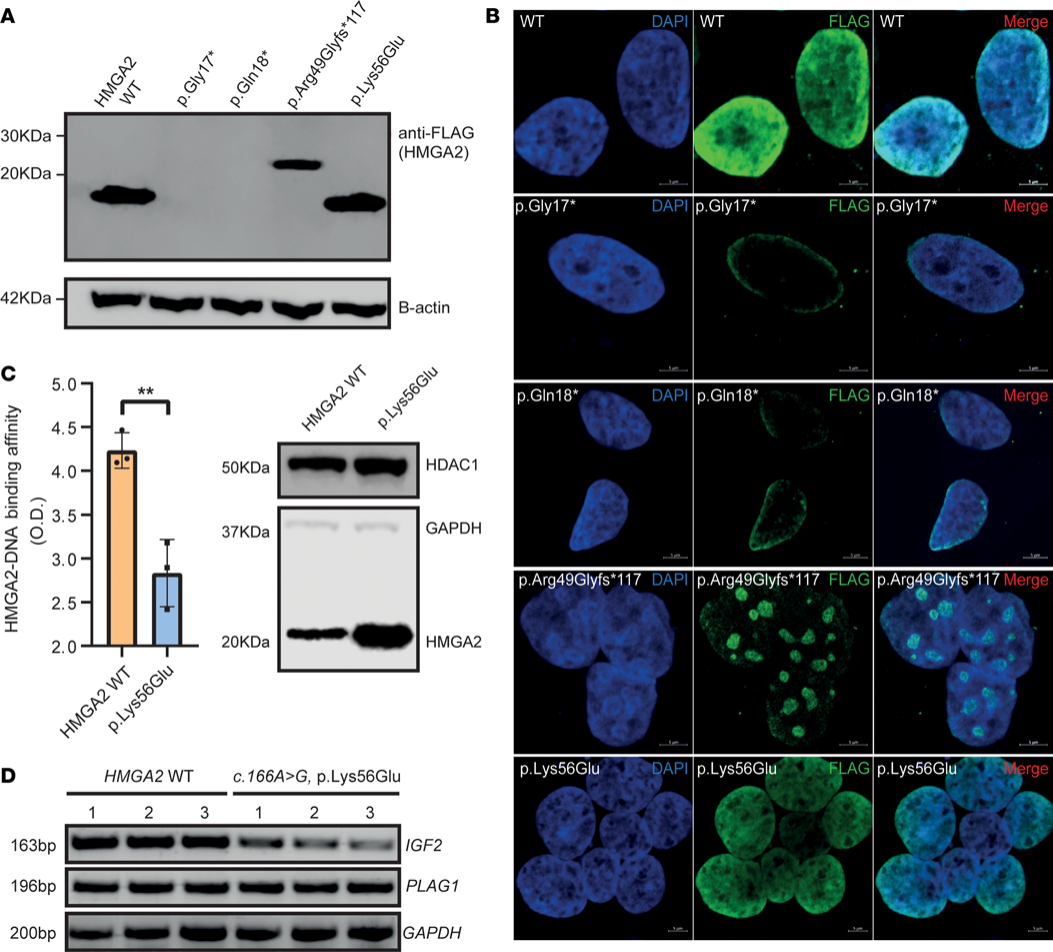
HMGA2 in vitro reconstitution experiments reveal altered variant protein expression and DNA binding activity. (**A**) Immunoblot analysis of FLAG-tagged WT and variant constructs in HEK293T cells revealed absence of detectable HMGA2 for both truncating variants (p.Gly17*, p.Gln18*) and reduced expression of higher molecular weight protein, p.Arg49Glyfs*117. Contrastingly, missense variant p.Ly56Glu was well expressed. (**B**) Confocal microscopy confirmed findings generated by immunoblotting, but further revealed enrichment of nuclear speckles in HEK293T cells expressing p.Arg49Glyfs*117. Scale bars: 5 μm. (**C**) Nuclear extracts of HEK293T cells transiently transfected with HMGA2-WT and p.Ly56Glu variant constructs were used in a colorimetric DNA binding assay using a double-stranded AT-rich DNA oligonucleotide known to interact with HMGA2. The p.Lys56Glu variant showed attenuated binding to this AT-rich DNA oligonucleotide compared with HMGA2-WT. Western blot shows nuclear HMGA2 protein input; GAPDH and HDAC1 served as loading controls. Data were analyzed using a 2-tailed, unpaired *t* test and are representative of 3 independent experiments presented as mean ± standard deviation. **P < 0.01. (**D**) HEK293T transfectants expressing the p.K56E variant showed reduced mRNA expression of *IGF2* in semiquantitative RT-PCR, whereas *PLAG1* expression was unchanged. *GAPDH* served as loading control.

In vitro assessments of *HMGA2* variants in a HEK293T expression system revealed reduced protein expression for p.Arg49Glyfs*117, while the missense variant p.Lys56Glu demonstrated protein levels similar to WT HMGA2 (Figure 2A). Both truncated nonsense variants (p.G17* and p.Q18*) were undetectable. When compared with HMGA2-WT, a protein of greater mass was visualized for p.R49Gfs*117, consistent with extension of the reading frame (Figure 2A). Immunofluorescence analyses of FLAG-tagged constructs showed nuclear localization for the p.Lys56Glu missense variant and lack of protein for the highly truncated p.Gly17* and p.Gln18* variants. Interestingly, the p.R49Gfs*117 variant demonstrated enhanced nuclear speckling (Figure 2B).

### Missense variant p.K56E alters DNA binding and IGF2 transcription.

Given the normal nuclear immunolocalization of the p.K56E variant when expressed in mammalian cells, we assessed the ability of p.Lys56Glu nuclear protein fractions to bind a specific biotinylated duplex oligonucleotide known to interact with HMGA2 (29). This variant, a missense variant occurring in linker 2, three amino acids distal to the 3′ end of AT-hook 2 domain, attenuated DNA-protein binding (Figure 2C), which may affect HMGA2 function. HMGA2 may alter growth via modulation of *IGF2* transcription either dependently or independently of PLAG1 (40). Transcript levels of *IGF2* and *PLAG1* were probed by RT-PCR following transfection of HMGA2-WT and p.Lys56Glu constructs into HEK293T cells. *IGF2* mRNA levels were reduced in the p.Lys56Glu variant compared with HMGA2-WT, whereas *PLAG1* transcript levels were unchanged (Figure 2D).

### Patient-derived fibroblasts demonstrate reduced HMGA2 expression and attenuated transcript levels of IGF2 and PLAG1.

**Figure 3:**
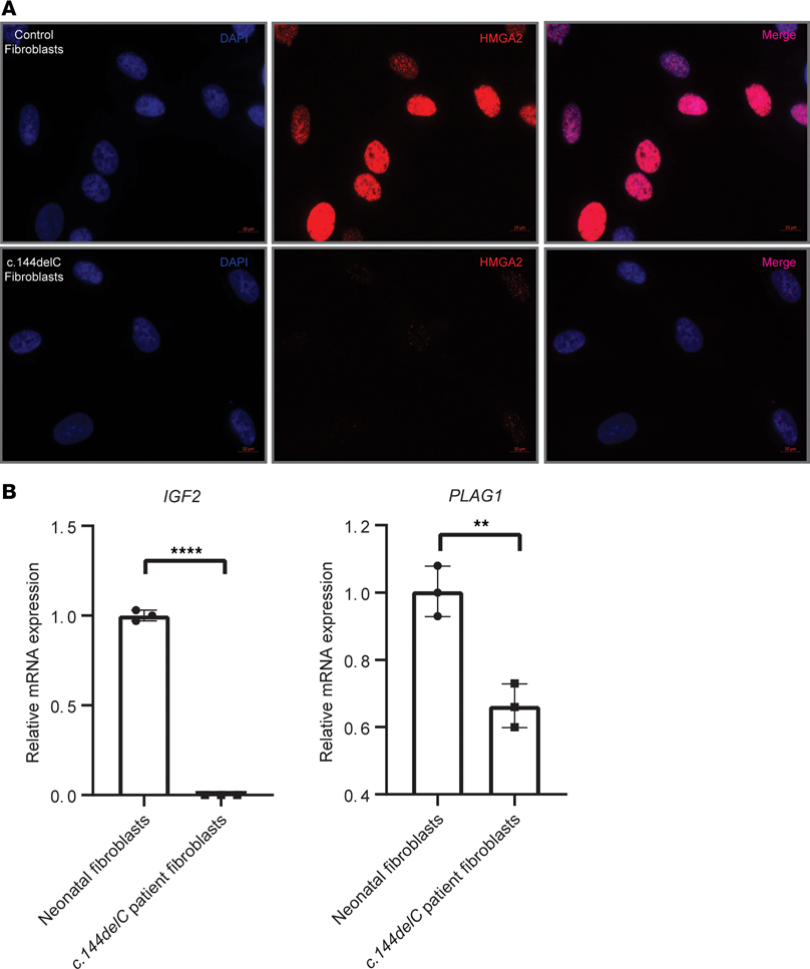
Patient-derived fibroblasts demonstrate attenuation of HMGA2 nuclear localization and *IGF2* transcription. (**A**) Neonatal control fibroblasts showed strongly positive nuclear HMGA2. Contrastingly, weak nuclear immunodetection of the c.144delC variant was observed in patient-derived fibroblasts. Original magnification, ×630. Scale bars: 10 μm. (**B**) Quantitative RT-PCR of c.144delC patient–derived fibroblasts showed abrogated mRNA expression of *IGF2* and reduced *PLAG1* expression when compared with control fibroblasts. Data were analyzed using a 2-tailed, unpaired *t* test and are representative of 3 independent experiments presented as mean ± standard deviation. **P < 0.01, ****P < 0.0001.

Dermal fibroblasts were cultured from a skin biopsy derived from patient 4 harboring the c.144delC (p.Arg49Glyfs*117) variant. Immunostaining of patient fibroblasts revealed weak detection of nuclear HMGA2 protein when compared with neonatal control fibroblasts (Figure 3A). Furthermore, *IGF2* mRNA transcript levels in patient fibroblasts were undetectable when compared with healthy control neonatal fibroblasts (Figure 3B). *PLAG1* levels were also decreased compared with healthy control neonatal fibroblasts, although to a lesser extent than *IGF2* (Figure 3B).

### Characterization of knockin mouse models.

**Figure 4:**
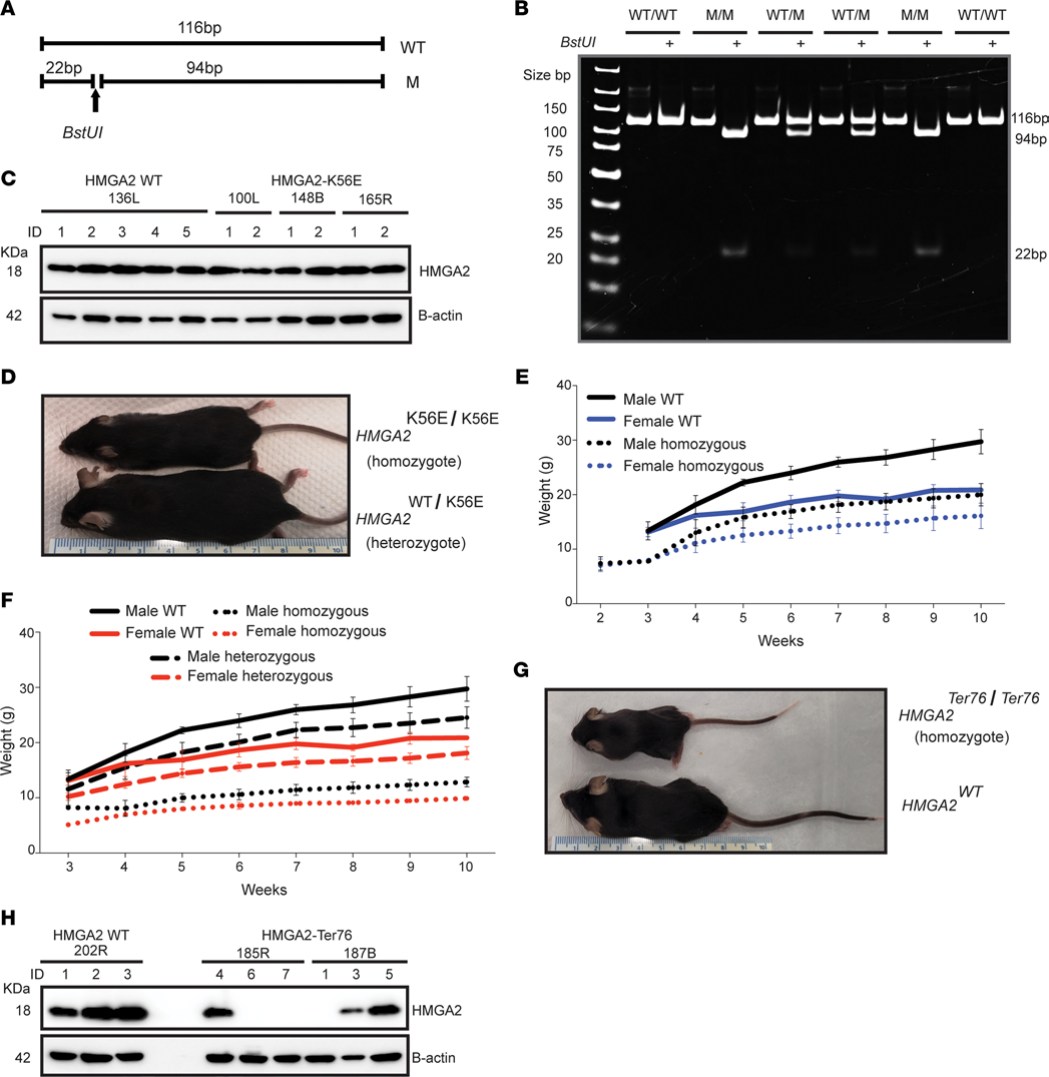
Generation of *Hmga2*-knockin mouse models. (**A**) Strategy for detection of the c.166A>G mutation; forward (5′-CCAGAGGAAGACCAAAAGGCCGC-3′) and reverse (5′-TGGAAACTTTACATGGAAGTCATTG-3′) primers were used to amplify the region surrounding the mutation. (**B**) Restriction enzyme digestion with *BstU*I followed by separation in a 10% polyacrylamide gel resulted in a 116-bp fragment for the WT and 94- and 22-bp fragments for the mutant sequence. (**C**) Total protein extracts from mouse embryonic fibroblasts (MEFs) isolated from *Hmga2*^WT^ and homozygous *Hmga2*^K56E^ mouse embryos were probed for Hmga2 expression by immunoblotting. The *Hmga2*^K56E^ variant showed protein levels equivalent to *Hmga2*^WT^. (**D**) A male homozygous *Hmga2*^K56E^ mouse is shown to be demonstrably smaller than an age- and sex-matched heterozygote at 12 weeks of age. (**E**) Body weights of age- and sex-matched WT and homozygous *Hmga2*^K56E^ mice were obtained weekly until 10 weeks old. Homozygotes consistently weighed less than WT counterparts. Male K56E, *n* = 47; female K56E, *n* = 44; male WT, *n* = 7; female WT, *n* = 7. Graphs were plotted using GraphPad Prism 9 software. (**F**) Heterozygous *Hmga2^Ter76^* mice demonstrated an intermediate growth-restricted phenotype, with lower weights when compared with age- and sex-matched WT mice. Homozygous mice were consistently smaller that both WT and heterozygotes. Male homozygous *Hmga2^Ter76^*, *n* = 11; female homozygous *Hmga2^Ter76^*, *n* = 3; male heterozygous *Hmga2^Ter76^*, *n* = 10; female heterozygous *Hmga2^Ter76^*, *n* = 12; male WT, *n* = 7; female WT, *n* = 7. Graphs were plotted using GraphPad Prism 9 software. (**G**) Male *Hmga2*^WT^ mouse and homozygous *Hmga2^Ter76^* mutant counterpart at 8 weeks of age. The homozygote showed a pygmy phenotype and was infertile. (**H**) MEFs isolated from embryos of heterozygous *Hmga2^Ter76^* breeders revealed an 18-kDa Hmga2 protein band (MEF 3,5-187B) similar to WT (MEF 1,2,3-202R and MEF 4-185R). Fibroblasts from homozygous *Hmga2^Ter76^* mice did not express Hmga2 (MEF 6,7-185R and MEF 1-187B).

CRISPR/Cas9 technology was utilized to generate heterozygous and homozygous knockin mice (*Hmga2*^K56E^) harboring the c.166A>G (p.Lys56Glu) variant observed in patient 1. *BstU*I restriction enzyme digestion of genomic DNA confirmed the A>G transition leading to this single amino acid substitution uniquely localized to the linker 2 region between AT-hook 1 and AT-hook 2 (Figure 4, A and B). Mouse embryonic fibroblasts (MEFs) derived from homozygous *Hmga2*^K56E^ mice expressed the mutated protein at similar levels to *Hmga2*^WT^ fibroblasts (Figure 4C). Compared with heterozygous age- and sex-matched littermates, homozygous *Hmga2*^K56E^ mice were fertile but SGA (Figure 4, D and E). Unlike the human condition, heterozygous *Hmga2*^K56E^ mice were not growth restricted. Occasionally, in homozygous *Hmga2*^K56E^ mice, dwarfism was associated with dysmorphic facial features, but this phenotype was inconsistent and mainly observed in young animals. Ongoing work involves investigating the molecular determinants of this developmental facial phenotype.

In the process of creating the *Hmga2*^K56E^-knockin mouse model, additional mice with deletions and insertions due to nonhomologous end joining repair were generated. One of these mice, with a clear pygmy phenotype, had a 14-bp nucleotide deletion (c.180–193delctctaaagcagccc) in *Hmga2* that resulted in a frameshift and introduction of a premature termination codon, confirmed by Sanger sequencing. The frameshift in the *Hmga2^Ter76^* mutation affects the proline at position 60 and leads to a premature termination at amino acid position 76. This results in reduction of linker 2 and omission of AT-hook 3 and the acidic C-terminus of Hmga2. In contrast with *Hmga2*^K56E^ heterozygotes, heterozygous *Hmga2^Ter76^* mice showed an intermediate growth-restricted phenotype when compared with age- and sex-matched WT counterparts (Figure 4F). Homozygous *Hmga2^Ter76^* mice were infertile and consistently showed a pygmy phenotype (Figure 4, F and G). MEFs derived from homozygous *Hmga2^Ter76^* embryos lacked detectable full-length Hmga2 protein expression (Figure 4H).

### MEFs from transgenic mice have reduced adipogenic potential.

**Figure 5:**
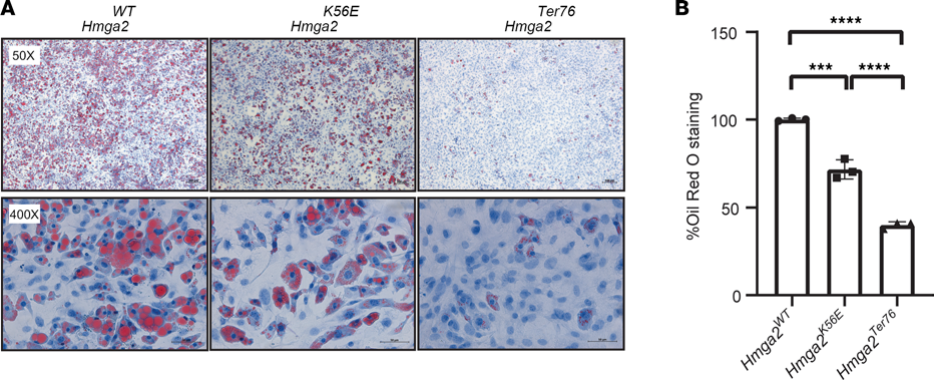
Adipogenic differentiation of mouse embryonic fibroblasts (MEFs). (**A**) *Hmga2*^WT^, *Hmga2*^K56E^, and *Hmga2^Ter76^* MEFs were seeded and adipogenic differentiation was induced. Lipid droplets were stained with Oil Red O and representative microscopic images at ×50 (top) and ×400 (bottom) magnification are shown. When compared with WT, mutants demonstrated reduced lipid droplet numbers and relative sizes. Scale bars: 200 μm (top) and 50 μm (bottom). (**B**) Quantification of stained lipid droplets was performed by eluting Oil Red O stain followed by absorbance measured at 510 nm. Data were analyzed using an ordinary 1-way ANOVA followed by Tukey’s test and are representative of 3 independent experiments presented as mean ± standard deviation. ***P < 0.001; ****P < 0.0001.

MEFs derived from WT and transgenic knockin homozygotes were differentiated into adipocytes in vitro. *Hmga2*^K56E^ and *Hmga2^Ter76^* MEFs demonstrated diminished adipogenic differentiation, as evidenced by a reduction in lipid droplet formation (Figure 5A) visualized by Oil Red O staining in *Hmga2*^K56E^ and *Hmga2^Ter76^* mutants compared with WT (Figure 5B).

## Discussion

We report 5 patients with pathogenic heterozygous variants in *HMGA2*. These cases presented with short stature and a spectrum of clinical features revealing the wide phenotypic, biochemical, and genetic landscape of this rare syndrome. Structure-phenotype correlation of our 5 variants suggests little difference in SRS severity among our patients, who all scored 3–4 out of 6 NH-CSS criteria and demonstrated comparable postnatal growth restriction. Despite reports of incomplete penetrance associated with variants in *HMGA2* (9), height segregated with maternal inheritance in patients 1, 3, and 5, where parental genotyping was possible. Our cohort corroborates reported clinical data suggesting that patients harboring *HMGA2* variants show features of SRS and a weak association with reduced head circumference rather than the macrocephaly typically observed in classical SRS (9). Knockdown of *Hmga2* in murine neuroepithelial cells has been shown to disrupt neurogenesis and neocortical development (50). However, *Hmga2*-knockout mice did not reveal reduced brain size, despite reduced body size, thus showing an allometric growth reduction (51).

Our cohort of patients with *HMGA2* variants included 2 nonsense variants, identified in patients 3 (c.52C>T, p.Gln18*) and 5 (c.49G>T, p.Gly17*), predicted to result in a premature termination prior to sequences encoding the first AT-hook. No HMGA2 protein was detected by immunoblotting following transient expression of these 2 variants in mammalian cells. The early predicted truncation of these variants may result in both transcripts being subject to nonsense-mediated mRNA decay, suggesting an association between haploinsufficiency of *HMGA2* and clinical SRS. This is consistent with the growth retardation phenotype in heterozygous *Hmga2*-knockout mice (42, 43) and with a CRISPR/Cas9 mouse model that produced a variant Hmga2 protein lacking functional AT-hooks 2 and 3, all linker regions, and the C-terminus (51), indicative of a functional *Hmga2* knockout.

Patient 2 (c.145delA, p.Arg49Glyfs*117) and patient 4 (c.144delC, p.Arg49Glyfs*117) variants resulted in a reading frame extension encoding the same protein. This larger protein is likely nonfunctional due to disruption of AT-hooks 2 and 3, but residual WT activity may be possible. Dermal fibroblasts derived from patient 4 (c.144delC) demonstrated a reduction in detectable nuclear HMGA2. The molecular genesis and structure of these 2 variants are distinctly different from a growing list of oncogenic HMGA2 fusion proteins that arise from chromosomal rearrangements in 12q14–q15 and maintain their ability to bind DNA and promote tumorigenesis (52–54). The function of these large HMGA2 proteins requires further investigation. Our preliminary findings suggest that exogenous overexpression of p.Arg49Glyfs*117 in HEK293T cells leads to enrichment of nuclear speckles detected by confocal microscopy. This variant may affect posttranscriptional splicing, leading to the accumulation of aberrant transcripts (55, 56), although the exact composition of these speckles remains to be determined.

The single amino acid substitution c.166A>G (p.Lys56Glu) variant is particularly interesting, since it is the first heterozygous missense variant to our knowledge affecting the HMGA2 linker 2 region identified in a patient with growth failure and SRS features. Unlike the other 4 *HMGA2* variants in our cohort, the p.Lys56Glu variant has functional AT-hooks and its nuclear localization was not impaired. In contrast with previously reported missense variants (p.Arg75Trp, p.Pro80Leu) located in AT-hook 3 (9, 41), this variant was located in linker 2, a region critical for HMGA2 protein-protein interaction (57, 58). Mutational Lys/Glu and Glu/Lys residue changes have been reported to alter protein-DNA binding and affect DNA maintenance and repair (59, 60). Our in vitro DNA binding assay revealed an attenuation of DNA binding of the p.Lys56Glu variant when compared with HMGA2-WT, suggesting an effect on nuclear HMGA2 function.

Previous work has indicated that the nonimprinted *HMGA2* can affect the expression of the imprinted *IGF2* gene, either dependently or independently of *PLAG1* (40). The human p.Lys56Glu variant construct expressed in HEK293T cells and patient-derived fibroblasts harboring the c.144delC variant both demonstrated reductions in *IGF2* mRNA transcript levels, whereas *PLAG1* expression was only reduced in the c.144delC patient fibroblasts. These data suggest an involvement of HMGA2 in *IGF2* gene expression in a PLAG1-independent manner.

To further address the functional impact of the p.Lys56Glu missense variant, we generated an *Hmga2*^K56E^-knockin mouse model. Homozygous *Hmga2*^K56E^ mice demonstrated a growth retardation phenotype and highlighted the importance of the Lys56 residue for HMGA2 functionality. However, heterozygous *Hmga2*^K56E^ mice were not small. Interestingly, a missense *HMGA2* variant in exon 3, c.239C>T, which leads to an exchange of proline to leucine at protein position 80 (p.Pro80Leu) in AT-hook 3, caused an SRS phenotype and severe growth restriction in 2 homozygous siblings, whereas heterozygous parents only showed slightly reduced growth (9). Our *Hmga2^Ter76^* mouse model demonstrated a growth-restricted phenotype in heterozygosity. The resultant frameshift would result in a protein with an N-terminally shortened linker 2 and absent AT-hook 3 and C-terminal domain. *Hmga2^Ter76^* homozygotes revealed a pygmy phenotype, suggesting that the extent of growth restriction directly correlated with the presence or absence of functional AT-hook 3 and/or the C-terminus. In contrast with smaller-sized homozygous *Hmga2*^K56E^ mice, haploinsufficiency of *Hmga2*^K56E^ failed to produce a growth-restricted phenotype, suggesting that the presence of a single copy of WT *Hmga2* could rescue the growth phenotype.

A common phenotypic feature seen in both human and murine HMGA2 deficiency models is reduced body weight. We demonstrated that both *Hmga2*^K56E^ and *Hmga2^Ter76^* homozygotes consistently weighed less than WT counterparts and corresponding MEFs had reduced adipogenic differentiation potential. *Hmga2* has been shown to be crucial for preadipocyte proliferation and adipogenesis, with *Hmga2* gene silencing resulting in the attenuation of adipocyte maturation and overexpression contributing to a murine obesity phenotype (61–63). Body composition data on pediatric SRS patients are sparse; however, Smeets et al. demonstrated that basal lean body mass and fat mass were both reduced in 29 SRS patients when compared with non-SRS individuals (64). Most patients had classical hypomethylation aberrations related to 11p15LOM and mUPD7. Feeding difficulties, poor weight gain, and hypoglycemia frequently seen in human patients may be countered by targeted manipulation of genetic targets and pathways affiliated with HMGA2-induced adipocyte formation. However, further work is needed to characterize the impact of monogenic SRS defects on body composition profiles and fat metabolism.

Three of the 5 patients harboring *HMGA2* mutations were treated with human GH (hGH) therapy, with variable responses. All 3 patients had short stature at the start of hGH treatment (–2.9, –4.1, and –3.1 SDS at ages 9.8, 7.5, and 5.5 years, respectively). Following approximately 5 years of treatment, patients 4 and 5 had modest height increases of +1 to +1.2 SDS and hGH therapy is on-going in patient 4. In contrast, despite a modest initial response, the final height of patient 3 was disappointing (–4.3 SDS). SRS is associated with earlier-onset puberty and gonadotropin-releasing hormone (GnRH) analogues (GnRHas) are recommended for at least 2 years in children with evidence of central puberty (starting no later than age 12 years in girls and 13 years in boys) to preserve adult height potential. GnRHa therapy was given to all 3 patients but only for 12 months in patient 3. These limited data suggest that responses to hGH therapy are poor or modest. Earlier onset of therapy in combination with GnRHa for at least 2 years at the appropriate age may improve the treatment responses. Compliance with therapy was not documented, so this may have contributed to poorer outcomes, especially in patient 3. More long-term prospective data are required to evaluate the efficacy of hGH treatment in SRS patients with monogenic causes. Targeted therapies geared toward ameliorating dysregulated signaling pathways may be useful in the future.

The pleiotropic nature of variants in *HMGA2* complicates delineation of genotype-phenotype correlations since mutation type often does not predict SRS phenotypic presentation. However, microcephaly appears to be a highly penetrant and consistent feature in SRS-like patients harboring pathogenic variants in *HMGA2*. The newly identified *HMGA2* mutations associated with SRS and the growth retardation phenotypes of our knockin mouse models strongly suggest that the relative spatial positioning between AT-hooks affects DNA binding and select functionality of HMGA2, as determined for adipogenic potential. In undiagnosed patients with clinical features of SRS but negative molecular/genetic analysis, *HMGA2* should be included in next-generation sequencing testing approaches.

## Methods

### Sex as a biological variable.

Sex was not considered as a biological variable for genetic analysis and the human skin fibroblast, MEF, and adipogenic differentiation experiments. Female and male mice were used to establish *Hmga2*-knockin mutant mice. Female and male mice were used for weight monitoring of WT, homozygous, and heterozygous offspring.

### Clinical and biochemical assessment.

Birth weight, height, and body mass index (BMI) values are expressed as SDS according to the appropriate Dutch or UK-WHO growth national standards. IGF-I levels are expressed as SDS based on age- and sex-appropriate ranges provided by the referral centers.

### Genetic analysis.

A total of 3500 short-stature patients were referred for diagnostic genetic analysis to the UK and Dutch centers. Patients with clinical suspicion of SRS (≥3 out of 6 NH-CSS criteria) underwent testing for SRS as first line. Patients negative for 11p15LOM and mUPD7 underwent whole-exome and/or targeted-genome sequencing. Genomic DNA was isolated from peripheral blood leukocytes using Qiagen DNeasy kits and the JANUS chemagic 360 Pro Workstation (PerkinElmer). In the UK, genetic variants were identified using custom bioinformatic pipelines that filtered genetic data generated from a whole-genome short-stature gene panel and whole-exome sequencing. The custom gene panel included entire genomic sequences of 65 growth disorder genes and 4 noncoding regions of interest, including 2000 bases upstream and 500 bases downstream. Probe design, preparation of libraries, capture, and sequencing were performed by Otogenetics Corporation. Sequencing was performed using an Illumina HiSeq 2500 platform. Variant call files were uploaded to Ingenuity variant analysis (IVA) (65) and data compared to a reference genome as previously described (65).

Dutch exomes were captured using the SureSelectXT Human all Exon v5 or Clinical Research Exome v2 capture library kit (Agilent Technologies) accompanied by paired-end sequencing on the HiSeq 4000 or NovaSeq 6000 (Illumina), generating 2 × 150 bp paired-end reads with at least 80× median coverage. An in-house sequence analysis pipeline, (Modular GATK-Based Variant Calling Pipeline, MAGPIE) based on read alignment using Burrows-Wheeler Alignment (BWA-MEM) and variant calling using the Genome Analysis Toolkit (GATK) Haplotype Caller and UnifiedGenotyper (66), was used to align reads and call variants on the generated BAM files. Variants were subsequently annotated using the Variant Effect Predictor (67). Included annotation fields were, among others, variant consequence, in silico prediction scores, and allele frequencies in the 1000 Genomes populations. An in-house-developed tool additionally annotated variants using dbSNP132, gnomAD, and the Genome of the Netherlands (GoNL) frequencies. After annotation, the data were filtered against a gene panel that consisted of 109–119 genes associated with short stature and variants with an allele frequency of greater than 5% in the GoNL or in the 1000 Genomes project were excluded. LOVDplus (Leiden Genome Technology Center, LUMC, Leiden) was used for interpretation of variants.

### HMGA2 variant sequencing and protein structure modeling.

*HMGA2* variants found on next-generation sequencing were confirmed by Sanger sequencing and evaluated using a combination of predictive tools: Sorting Intolerant from Tolerant (68), Polymorphism Phenotyping v2 (69), and MutationTaster (70). Protein 3D modeling of the Alpha Fold Protein Structure Database (71) HMGA2 crystal structure AF-P52926-F1 model was performed using PyMOL v2.3.3 (https://pymol.org/2/) and IntFOLD Integrated Protein Structure and Function Prediction Server (72).

### Site-directed mutagenesis and generation of HMGA2 constructs.

Site-directed mutagenesis of an N-terminally FLAG-tagged *HMGA2* (NM_003483.4) human ORF clone was performed using the QuikChange II XL site-directed mutagenesis kit (Agilent, 200521) according to the manufacturer’s instructions. Primers for generation of 3 single nucleotide substitution variants (c.49G>T, c.52C>T, and c.166A>G) were designed using the online tool https://www.agilent.com/store/primerDesignProgram.jsp The frameshift construct was customized by GenScript to recapitulate reading frame extension and generation of a prolonged protein.

### Cell culture, transfection, and nuclear fractionation.

HEK293T (ATCC, CRL-3216), human skin fibroblasts (ATCC PCS-201-012), and MEFs (isolated from day 13.5 embryos) were cultured in high-glucose DMEM supplemented with 10% FBS and 1% penicillin/streptomycin and grown at 37°C in 5% CO_2_. *HMGA2*^WT^ and variant plasmid constructs were transfected into HEK293T cells using Lipofectamine 3000 (Invitrogen) according to the manufacturer’s instructions. Nuclear and cytoplasmic cell fractions were prepared using NE-PER Nuclear and Cytoplasmic Extraction Reagents (Thermo Fisher Scientific) according to the manufacturer’s instructions.

### Immunofluorescence.

Cells seeded on glass coverslips (24-well plate) were fixed with 4% paraformaldehyde for 15 minutes. Cells were then washed 3 times in PBS and permeabilized in ice-cold 100% methanol for 10 minutes at –20°C. After 3 further PBS washes, coverslips were incubated in Blocking buffer (1× PBS, 5% goat serum, and 0.3% Triton X-100) at room temperature for 60 minutes. Primary antibodies monoclonal anti-FLAG M2 (Sigma-Aldrich, catalog F3165) and anti-HMGA2 (Cell Signaling Technology, catalog 5269) reconstituted in dilution buffer (1× PBS, 1% BSA, 0.3% Triton X-100) was added to cells and left at 4°C overnight with gentle agitation. Cells were then washed 3 times with PBS prior to addition of fluorescent secondary antibody (goat anti–mouse IgG [H+L] cross-adsorbed secondary antibody Alexa Fluor Plus 488, A32723; and goat anti–rabbit IgG [H+L] highly cross-adsorbed secondary antibody, Alexa Fluor Plus 594, A32740; both Thermo Fisher Scientific) and left at room temperature for 90 minutes (protected from light). Coverslips were counterstained with DAPI and washed with PBS prior to mounting on microscope slides.

### DNA binding assay.

HMGA2-DNA binding was assessed using the commercially available DNA-protein binding colorimetric assay kit (Abcam, ab117139) according to the manufacturer’s instructions. Nuclear extracts were prepared from HEK293T cells transfected with HMGA2-WT and p.Lys56Glu constructs. Nuclear HMGA2-WT and p.K56E extracts (10 μg each) were incubated with a 50-bp biotin-labeled duplex oligonucleotide (5′-biotin-TEG-TTTTACGTTTCTCGTTCAGCTTTTTTATACTAACTTGAGCGAAACGGGAA-3′ and 5′-TTCCCGTTTCGCTCAAGTTAGTATAAAAAAGCTGAACGAGAAACGTAAAA-3′) and subsequently exposed to 1 μg/mL anti-HMGA2 antibody. Goat anti–rabbit IgG H&L (HRP) (Abcam, catalog ab205718) was used as the secondary antibody and binding evaluated by absorbance measured at 450 nm using a microplate reader.

### Generation of Hmga2^K56E^- and Hmga2^Ter76^-knockin mice.

*Hmga2*-knockin mutant mice are listed as “Hokl” under the laboratory registry code and were generated by CRISPR/Cas9 gene editing at the University of Manitoba Transgenic Services platform. To introduce the p.K56E mutation (NM_010441.3: c.166A>G, p.Lys56Glu) into mice, a guide (5′-CACCTTCTGGGCTGCTTTAG-3′) located downstream from the nucleotide to be modified was synthesized as an Alt-R CRISPR/Cas9 crRNA by Integrated DNA Technologies (IDT).

A single-stranded donor DNA (5′-AGCCAACCTGTGAGCCCTCTCCTAAGAGACCCAGAGGAAGACCAAAAGGCAGC**G**AAAACAAGAGCCCTTCTAAAGCAGCCCAGAAGGTGAGAATTCTCATGTCAAGTTCTT-3′) designed to introduce the desired substitution (bold) while also destroying sites for a second backup guide (c.156C>A, underlined) as well as the PAM site (c.180C>T, underlined) was synthesized by IDT. C57BL/6J zygotes generated by in vitro fertilization (73) were electroporated in 10 μL of Opti-MEM (Thermo Fisher Scientific) containing 500 ng/μL Cas9 (Alt-R S.p. Cas9 Nuclease V3), 200 ng/μL of the guide duplex (Alt-R CRISPR-Cas9 crRNA and tracrRNA), and 400ng/μL of ss DNA donor. Electroporation was done using the Bio-Rad Gene Pulser Xcell at 30 V, 1 second ON, 99 seconds OFF, for 12 cycles. Zygotes were cultured to the 2-cell stage and then transferred to CD1 pseudopregnant mice (0.5 dpc). To identify the c.166A>G substitution, a 116-bp region encompassing the mutation was PCR amplified. As shown in Figure 3, an A>C substitution 3 bp from the end of the forward primer created a CGCG sequence only in the presence of the desired c.166A>G mutation and recognized by the restriction enzyme *BstU*I. This strategy was used to demonstrate the presence of the K56E mutation in 12 of 44 offspring, which was confirmed by Sanger sequencing. Of these, 6 mice that did not appear to have any other mutations were used as initial breeders to study the phenotype associated with the K56E mutation (*Hmga2*^K56E^). At the same time, several founders with insertions and deletions in *Hmga2* due to nonhomologous end joining repair were identified. One of these founders was identified to have a 14-bp deletion that resulted in a frameshift and introduction of a premature termination translation codon after amino acid 76 (NM_010441.3: c.180–193delctctaaagcagccc, *Hmga2^Ter76^*).

### DNA extraction.

Mouse ear punches were incubated in DNA lysis buffer (100 mM Tris, pH 8.0, 200 mM NaCl, 5 mM EDTA, 0.2% sodium dodecyl sulfate, and 250 μg/mL proteinase K) at 55°C overnight. Samples were centrifuged at 18,500*g* for 10 minutes and the supernatants were transferred to a new tube. Isopropanol was used to precipitate DNA and DNA was pelleted through centrifugation at 18,500*g* for 5 minutes. ddH_2_O was used to dissolve the DNA pellet and DNA concentrations were determined by Synergy H1 using Take3 plates (BioTek).

### PCR and restriction enzyme digestion.

DNA (100 ng) was used to amplify the *HMGA2* gene using the following primers: WPG1265 5′-CCAGAGGAAGACCAAAAGGCCGC-3′ and WPG1266 5′-TGGAAACTTTACATGGAAGTCATTG-3′. Samples were denatured at 95°C for 5 minutes followed by 40 cycles of 95°C for 1 minute, primer annealing at 60°C for 1 minute, extension at 72°C for 1 minute, and a final extension at 72°C for 5 minutes. For detection of the K56E mutation, the PCR products were subjected to restriction enzyme digestion using 5 U of *BstU*I enzyme (5 μL of PCR product with 0.5 μL of *BstU*I), followed by incubation at 37°C for 3 hours in a PCR machine. The PCR products to detect p.60fs76 were not digested. The digested and undigested PCR products were loaded onto a 10% polyacrylamide gel before running at 100 V for 50 minutes. The gels were stained with 0.5 μg/μL ethidium bromide and visualized under UV light using a G:BOX Chemi XX6 (Syngene).

### MEF isolation.

The embryos were collected from day 13.5–14.5 pregnant mice according to a published protocol (74). Briefly, each embryo was separately processed by mincing to small pieces and further digested with trypsin for 40 minutes at 37°C. Complete medium (DMEM/F12 with 10% FBS and 1% pen/strep; Gibco, Thermo Fisher Scientific) was used to stop the trypsin reaction. Homogenization was achieved by pipetting up and down to break up the tissue. The cell suspension was plated to a new 15 cm petri dish and incubated at 37°C in a humidified incubator in 5% CO_2_ until cells were confluent.

### RT-PCR.

HMGA2-WT and p.Lys56Glu variant clones were transfected into mammalian HEK293T cells for 24 hours followed by RNA extraction. cDNA synthesis was performed using the High-Capacity cDNA Reverse Transcription Kit (Applied Biosystems) and RT-PCR conducted using gene-specific primers *IGF2* (Forward 5′-CGTGGCATCGTTGAGGAGTG-3′ and Reverse 5′-TGTCATATTGGAAGAACTTGCC-3′) and *PLAG1* (Forward 5′-TTCACTCCTACTCTCACACAG-3′ and Reverse 5′-GGGTCGTGTGTATGGAGGTG-3′). PCR products were analyzed on a 2% agarose gel.

### qPCR.

Total RNA extraction from human fibroblast cells was carried out using TRIzol reagent (Invitrogen, Thermo Fisher Scientific). cDNA synthesis was performed using 1 μg of RNA and qScript cDNA master mix (Quanta Biosciences). Quantitative real-time polymerase chain reaction (qPCR) was performed utilizing aforementioned human *IGF2* and *PLAG1* primers with amplification by PowerUp SYBR Green Master Mix (Applied Biosystems, Thermo Fisher Scientific). Gene expression analysis was performed by the comparative CT (ΔΔCT) method using QuantStudio Design & Analysis software. Samples were normalized to the expression of *GAPDH*.

### Adipogenic differentiation.

MEFs (6 × 10^4^) were cultured in 24-well plates for 24 hours. Subsequently, the culture medium was replaced with MesenCult adipogenic differentiation medium (StemCell Technologies) for 6 days. Following the differentiation period, cells were fixed using 4% paraformaldehyde for 15 minutes at room temperature. The fixed cells were then stained with Oil Red O for 30 minutes. To quantify the Oil Red O staining, 100% isopropanol was added, and cells were incubated on a shaker for 10 minutes to release Oil Red O from stained cells. The resulting Oil Red O solution in isopropanol (100 μL) was transferred to a 96-well plate and the absorbance measured at 510 nm.

### Immunoblotting.

Whole-cell lysates were prepared by addition of RIPA buffer (Sigma-Aldrich) supplemented with protease and phosphatase inhibitor tablets (Roche) and nuclear extracts prepared as above. Protein concentrations were quantified using a Bradford protein assay (Bio-Rad) and lysates denatured by addition of sodium dodecyl sulfate sample buffer 6× (Sigma-Aldrich) and boiled for 5 minutes at 98°C. A 20-μg bolus of protein was loaded into the wells of a 4%–20% sodium dodecyl sulfate–PAGE gel (Novex) prior to electrophoretic separation using MOPS buffer. Protein transfer to a nitrocellulose membrane was achieved by electroblotting at 15 V for 45 minutes. The membrane was blocked with 5% fat-free milk in Tris-buffered saline/0.1% Tween 20 (TBST) and left to gently agitate for 1 hour. Primary antibodies (anti-FLAG M2 and anti-HMGA2 antibody) were added at a dilution of 1:1000 with GAPDH and HDAC1 as housekeeping controls (rabbit anti-GAPDH antibody, Abcam, catalog ab9485; mouse anti-HDAC1 antibody, Santa Cruz Biotechnology, catalog sc-81598) at a concentration of 1:10,000. Primary antibody incubation was left overnight at 4°C with gentle agitation. The membrane was then washed for 5 minutes (3 times) with TBST. Secondary antibodies (IRDye 800CW goat anti–rabbit IgG; RRID: AB_10796098 and IRDye 680RD goat anti–mouse IgG; RRID: AB_2651128; both Li-COR Biosciences) were added at a dilution of 1:5000 in blocking buffer and the membrane incubated at 37°C for 60 to 90 minutes. The membrane was subsequently washed 3 times (5 minutes each) with TBST and visualized with the LI-COR Image Studio software for immunofluorescence detection.

For the analysis of MEFs, protein lysates were extracted using 1× Laemmli buffer, run in 12% sodium dodecyl sulfate–PAGE gels, and blotted onto nitrocellulose membranes. Nonspecific protein binding sites were blocked by incubating with 5% fat-free milk in TBST for 60 minutes at room temperature before incubating with 1:1000-diluted rabbit anti-HMGA2 antibody at 4°C overnight. Membranes were washed 3 times (5 minutes each) with TBST then further incubated with HRP-conjugated goat anti-rabbit secondary antibody for 60 minutes at room temperature. β-Actin was used as a loading control. Precision Plus Protein All Blue Prestained Protein Standards (Bio-Rad) was used as standard to determine the molecular weight. Immunoreactive bands were visualized with ECL Clarity (Bio-Rad) using Bio-Rad Chemi-Doc MP Imagers.

### Statistics.

The experiments were done in triplicate. The results are represented as mean ± standard deviation. Statistical analysis was performed using GraphPad Prism 9 software with 1-way ANOVA and unpaired *t* tests. *P* values less than 0.05 were considered significant: *P < 0.05, **P < 0.01, ***P < 0.001, and ****P < 0.0001.

### Study approval.

Informed written consent for genetic research and publication of clinical details was obtained from patients (when 12 years or older) or their parents. The study was approved by the Health Research Authority, East of England-Cambridge East Research Ethics Committee (REC reference 17/EE/0178). The transgenic mouse work was approved by the animal ethics committee at the University of Manitoba (protocol 21-018).

### Data availability.

A Supporting Data Values file is included in the supplemental material. Other data are available from the corresponding author upon request.

## Author contributions

AVM, EC, and TT are co–first authors. Authorship order was determined by the level of contribution to the writing of the manuscript and generation/analysis of experimental data. TK, SHK, AVM, and HLS conceptualized the study. TK, SHK, VH, and HLS supervised the experimental work. AVM, EC, TT, and MF performed the experimental work and conducted data acquisition and analysis. SDJ, SGK, DVDK, ACDB, ASB, TR, GAA, MICCL, AM, HAVD, IMDE, and CDB collected clinical data and phenotyped participants. BTR, TT, TK, and SHK generated and characterized the transgenic mouse models. AVM conducted protein modeling. AVM, EC, and TT generated the initial manuscript. All authors contributed to critical appraisal and the final draft of the manuscript.

## Supplementary Material

Unedited blot and gel images

Supporting data values

## Figures and Tables

**Table 2:**
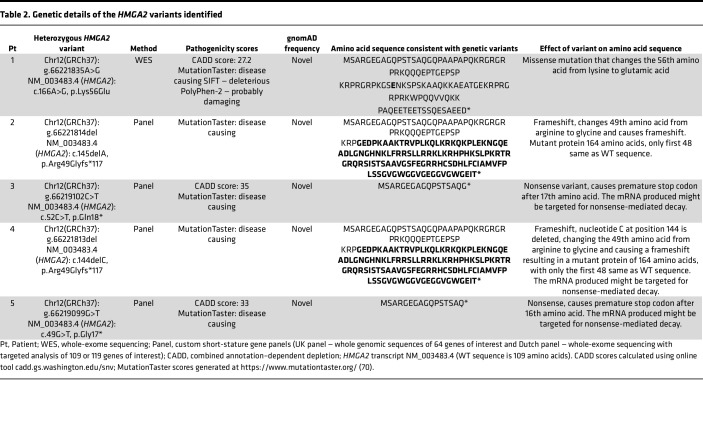
Genetic details of the *HMGA2* variants identified

**Table 1:**
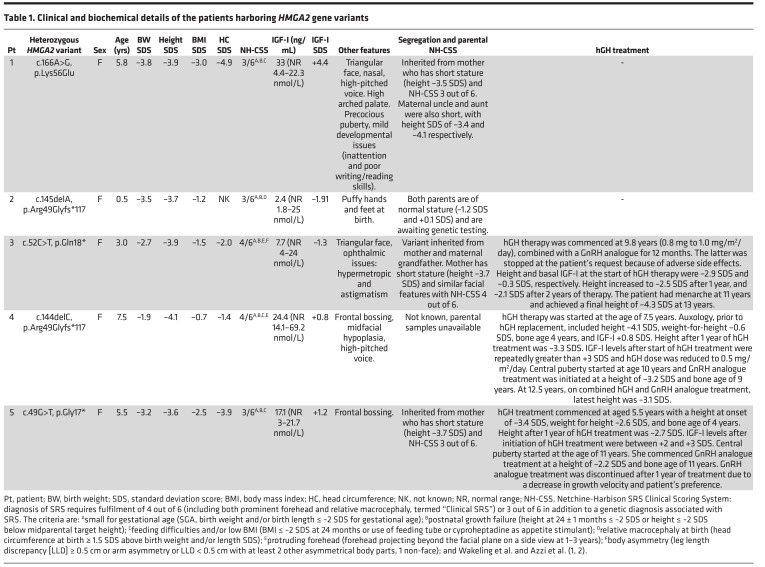
Clinical and biochemical details of the patients harboring *HMGA2* gene variants

## Version 1 03/22/2024

Electronic publication
