# Supplementary figures and images for: Characterization of HMGA2 variants expands the spectrum of Silver-Russell syndrome
Source: JCI Insight. 2024 Mar 22;9(6):e169425. doi: 10.1172/jci.insight.169425 (PMC11063932; doi:10.1172/jci.insight.169425)

Fig 2A

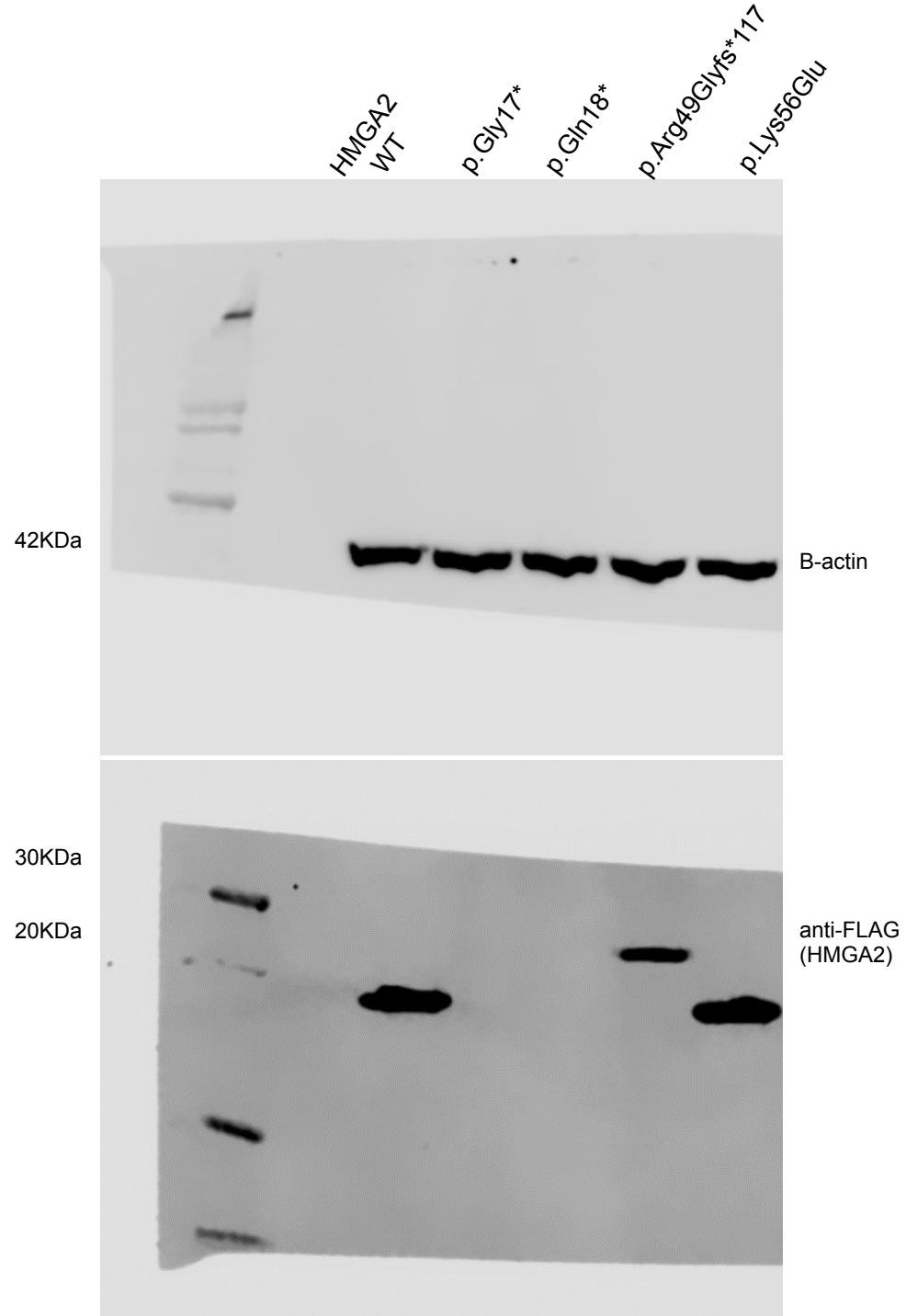

Fig 2C

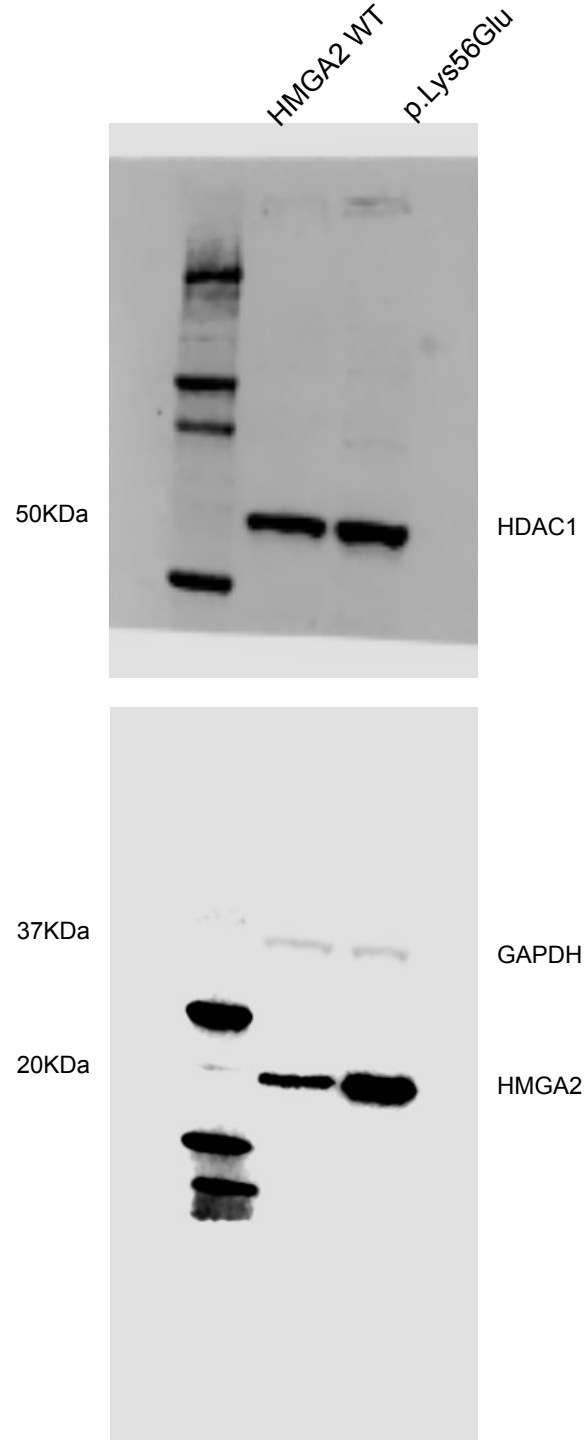

Fig 2D

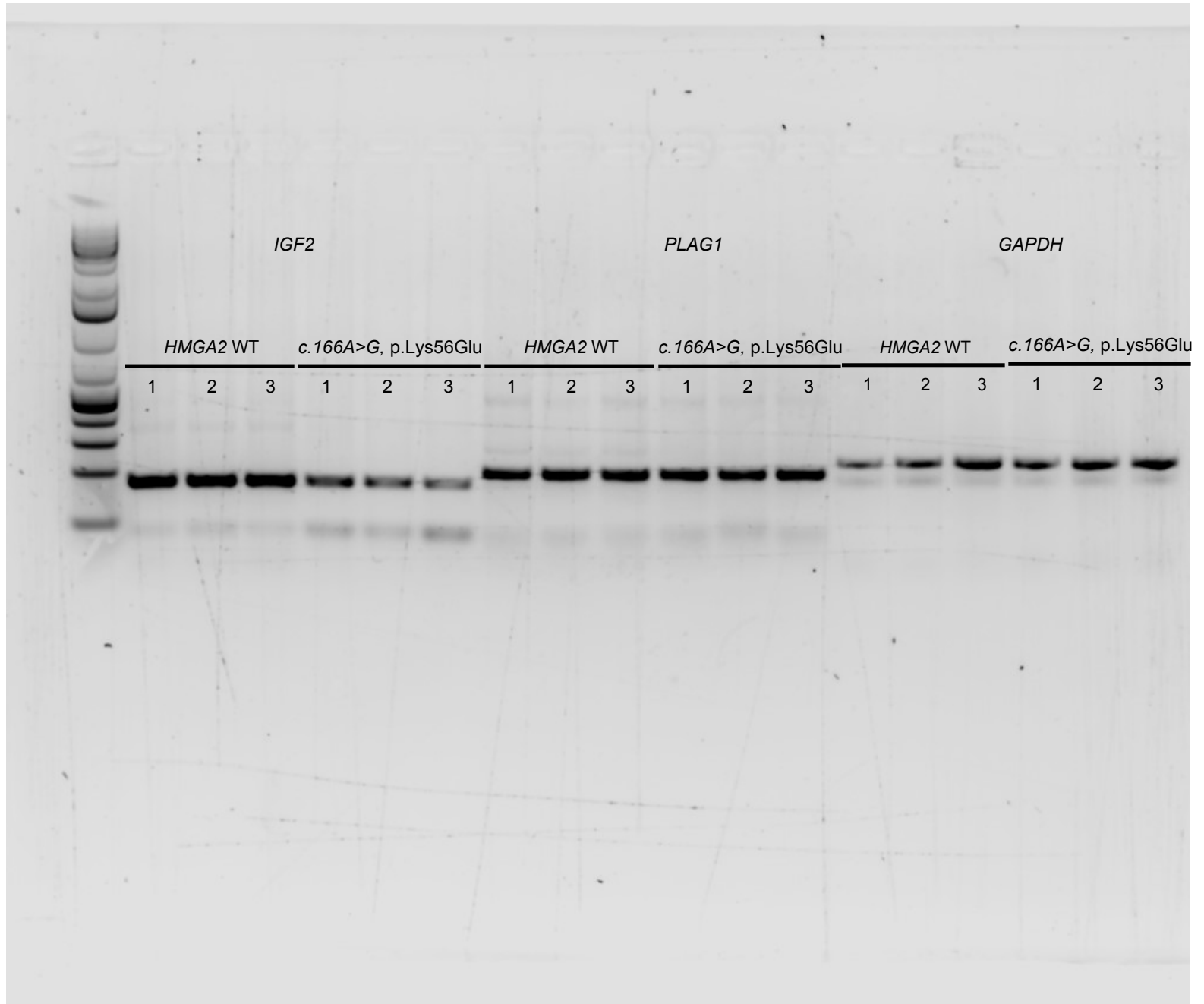

Fig 4B

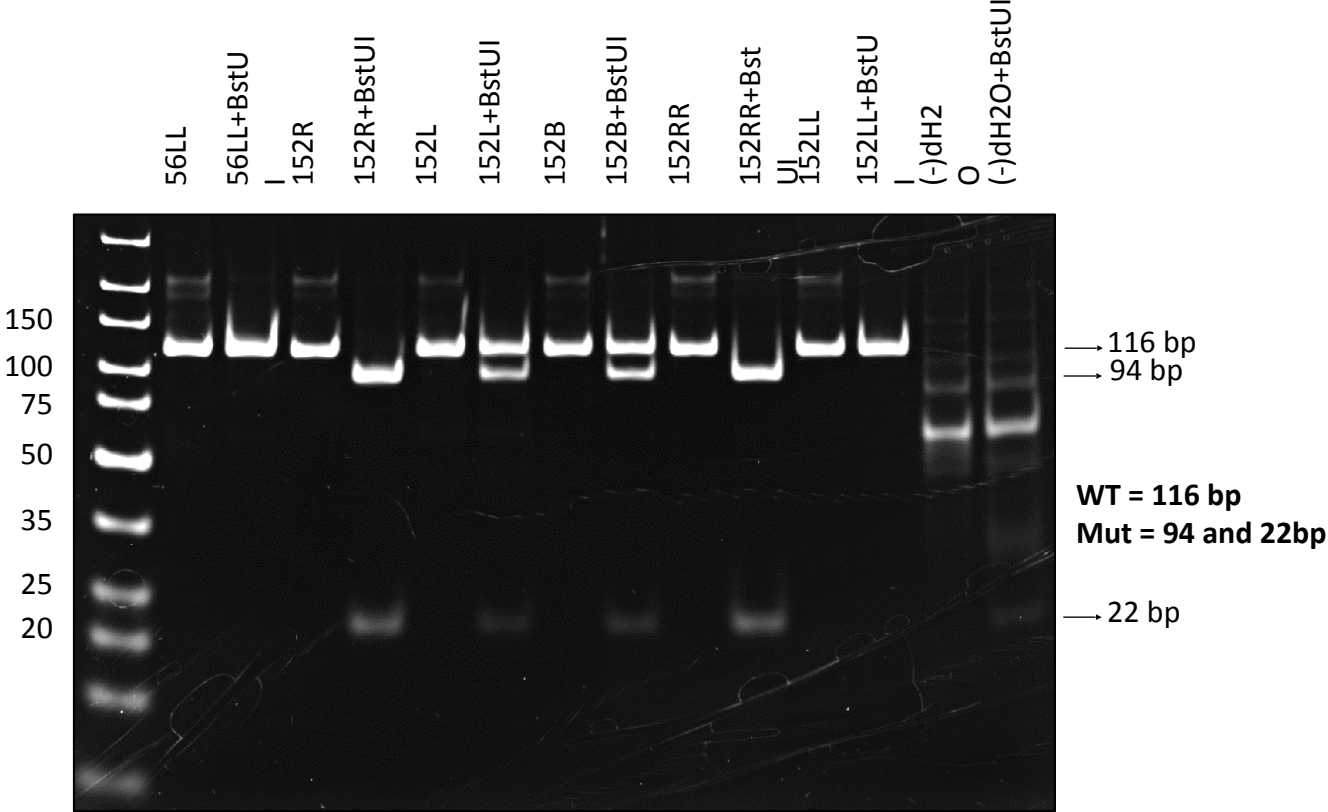

| HMGA2-WT |   |   |   |   | HMGA2-K56E |   |      |   |      |   |               |
|----------|---|---|---|---|------------|---|------|---|------|---|---------------|
| 136L     |   |   |   |   | 100L       |   | 148B |   | 165R |   |               |
| 1        | 2 | 3 | 4 | 5 | 1          | 2 | 1    | 2 | 1    | 2 |               |
|          |   |   |   |   |            |   |      |   |      |   | HMGA2, 18 kDa |

HMGA2, 18 kDa

Fig 4C

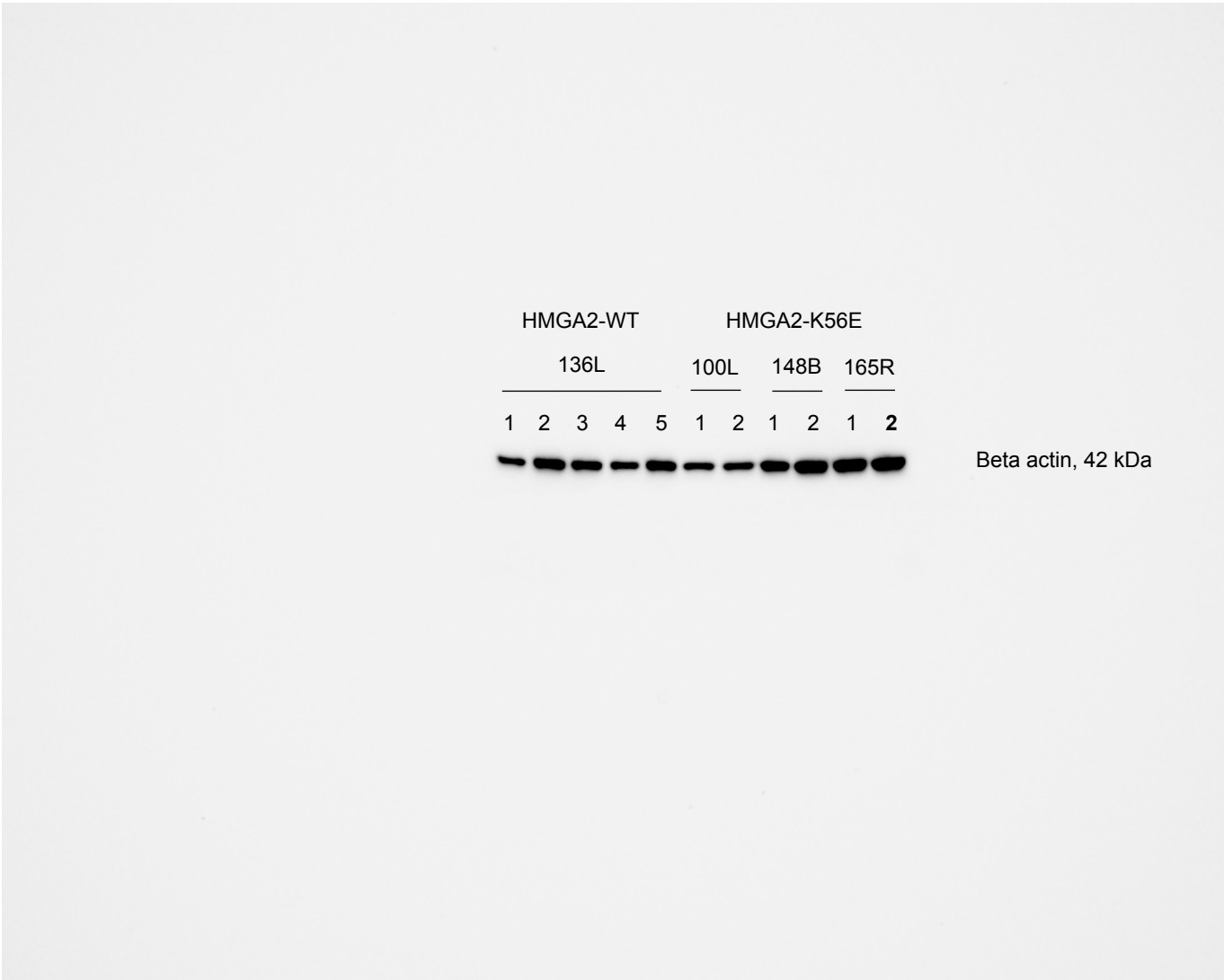

Fig 4H

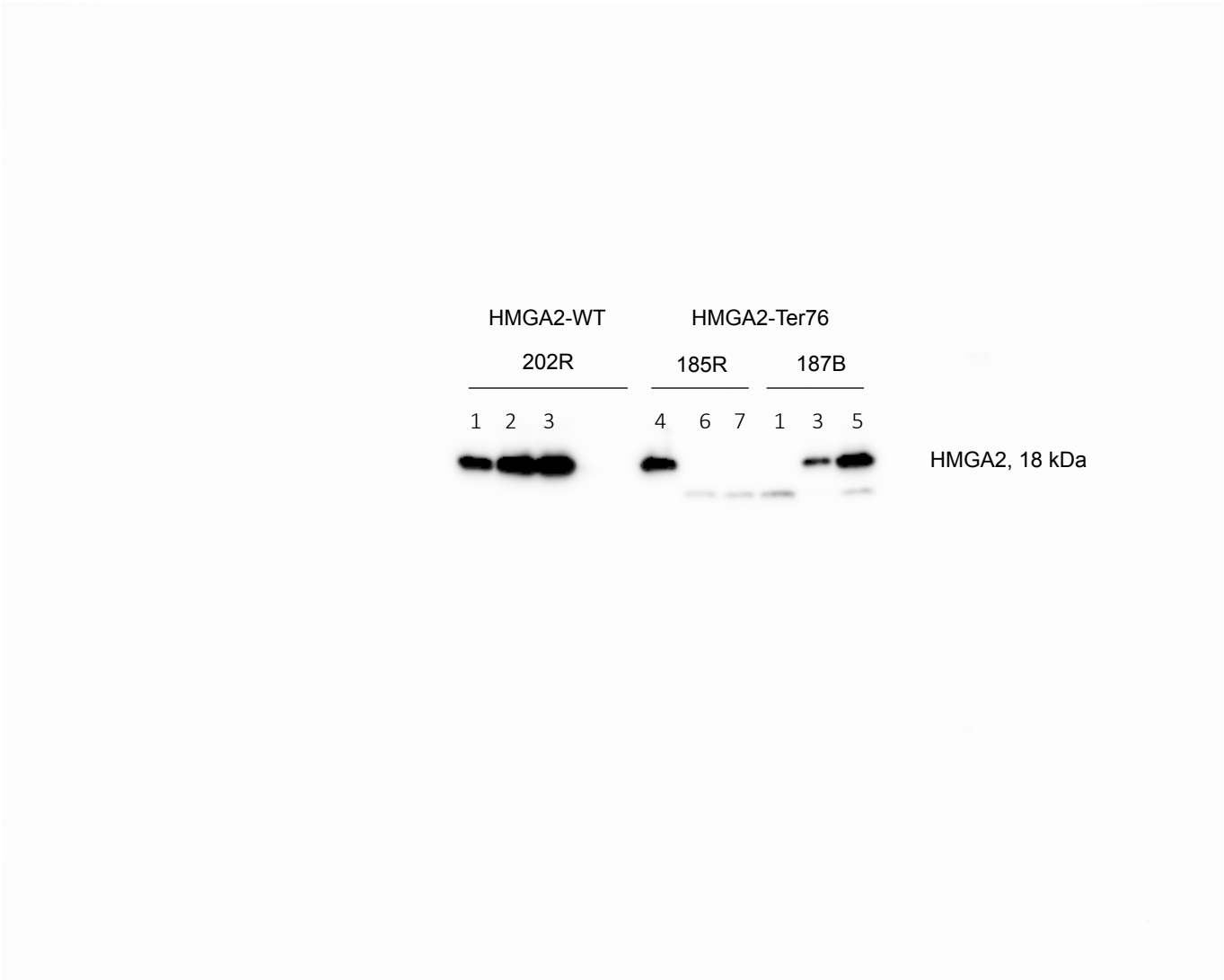

Fig 4H

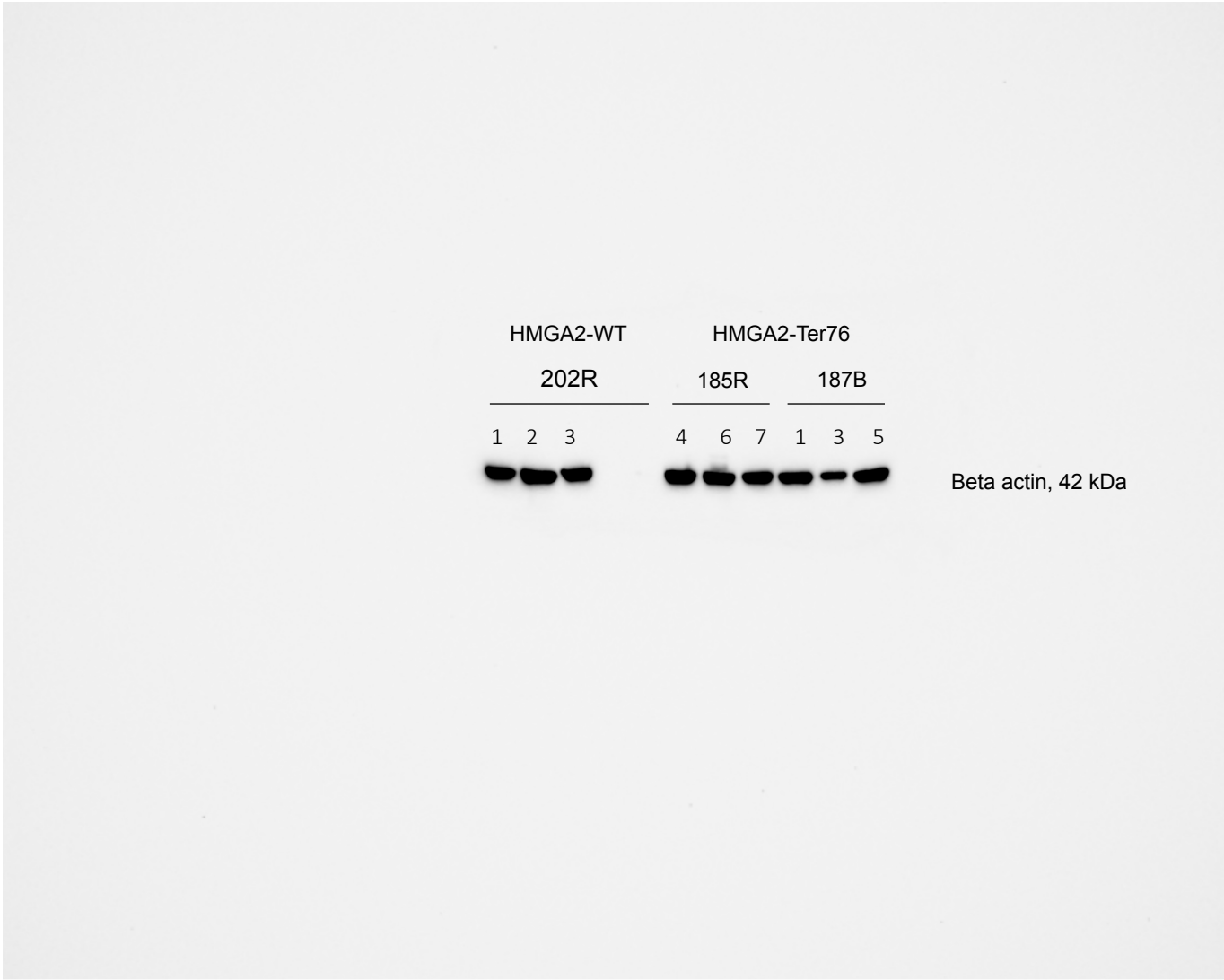

Supplement: Unedited blot and gel images [file jciinsight-9-169425-s118.pdf]
